# Communication Practices in Surgical Settings: A Qualitative Study of Healthcare Providers and patient Caregivers in Coastal Karnataka

**DOI:** 10.1177/11786329261474981

**Published:** 2026-08-29

**Authors:** Dnyata Pandit, Kirthinath Ballala, G. Somu, Anitha Nileshwar, T. Latha, Selim Jahangir, T. R. Aishwarya, Asifa Nikhat, Shivani Muneeswaran, Deepthi U. Hegde, J. Athira, Rajesh Kamath

**Affiliations:** 1 Department of Hospital Administration, 29224 Kasturba Medical College, Manipal Academy of Higher Education, Manipal, India; 2 Department of Community Medicine, 29224 Kasturba Medical College, Manipal Academy of Higher Education, Manipal, India; 3 Department of Anesthesiology, 29224 Kasturba Medical College, Manipal Academy of Higher Education, Manipal, India; 4 College of Nursing, 596687 AIIMS Bibinagar, Bibinagar, India; 5 Department of Social and Health Innovation, 636621 Prasanna School of Public Health, Manipal Academy of Higher Education, Manipal, India; 6 Department of Healthcare and Hospital Management, 636621 Prasanna School of Public Health, Manipal Academy of Higher Education, Manipal, India

**Keywords:** healthcare communication, caregiver, digital health, healthcare provider, perioperative care

## Abstract

**Background:**

Communication breakdowns in perioperative settings are a major contributor to elevated stress, heightened anxiety, and emotional vulnerability among patient caregivers, thus highlighting the need for reliable communication practices. This qualitative study investigated communication practices through the perceptions and experiences of patient caregivers and healthcare providers across the perioperative stages, exploring the benefits and challenges of both verbal and digital communication methods.

**Methodology:**

A qualitative research design was adopted, involving semi-structured in-depth interviews with 23 purposively selected participants from a tertiary hospital in Coastal Karnataka. The interview guides were pilot tested and validated to ensure accuracy and comprehensiveness. The interviews were audio-recorded, transcribed, and analyzed using ATLAS. ti 8 software for thematic analysis. A two-cycle coding process was employed to analyze the data from the participants’ perspectives, resulting in eleven different codes that were further organized into six emerging themes.

**Results:**

The study revealed existing communication practices in surgical settings, with verbal communication practices characterized by direct updates and mic announcements emerging as more prominent. However, limited intraoperative updates intensified patient caregiver anxiety and uncertainty. The participants perceived the benefits of digital adoption of communication tools to bridge the communication gaps in surgical settings, although concerns remained about digital literacy, data privacy, and over-reliance on technology.

**Conclusions:**

The successful implementation of digital communication practices depends on overcoming structural, socio-ethical, and technological limitations. Alternatively, a tailored communication approach that reflects stakeholder needs in peri-operative contexts would be more effective in sustainable communication delivery.

## 1. Introduction

The World Health Organization (WHO) has estimated that over 200 million surgeries are performed annually, and many surgical patients experience adverse events, despite surgical care being an essential component of healthcare services globally.^1^ Research highlights that communication errors are the major contributors to such adverse events, occurring approximately every 7-8 minutes in face-to-face interactions. These communication gaps are primarily related to cross-disciplinary exchanges, a lack of clarity and transparent communication practices, insufficient or inaccurate information sharing with patient caregivers, uncertainty about procedure timelines, and a lack of structured communication practices. These communication inefficiencies are compromising surgical care quality and patient safety. Moreover, the operating room is a highly complex and challenging environment, including three stages of perioperative care: preoperative, intraoperative, and postoperative care involving surgeons, anaesthesiologists, nurses, and allied health care professionals (HCPs).^2^ As per JCI, ineffective communication ranked as one of the top three causes of sentinel events between 1995 and 2005, and between 2010 and 2013 as well, accounting for 66% of all sentinel events. Recent reports of 2022 and 2023 confirm the same.^3-5^ Research indicates that a significant proportion of these adverse events and their associated morbidity and mortality are not related to technical incompetencies but rather to communication breakdowns in healthcare settings.^3-6^ Thus, improving the effectiveness of communication among patient caregivers has become one of the Joint Commission National Patient Safety Goals for Hospitals.^6,7^

Ideal and effective communication in the given surgical context refers to a clear, transparent, complete, timely, and consistent message from a sender to a recipient, encompassing both verbal and digital modes of communication. From pre-surgical optimisation to a smooth procedure and recovery in the post-anaesthesia care unit, communication underpins effective patient care in surgical settings.^8^ Studies demonstrate that communication failures contribute to more than 56% of intraoperative and postoperative complications, primarily resulting from verbal communication practices where either the information was not transmitted or inaccurately received from a single transmitter to a single receiver. This underscores the need for closed-loop communication practices.^8,9^ An Italian study analysed 1,235 patient and patient caregiver complaints that reported top verbal communication challenges, including receiving inaccurate information, inaccessibility to information, and inappropriate timeliness of updates.^10^

Globally, the surgical environment is acknowledged as a high-risk and high-pressure environment. Elevated levels of stress, distractions, organisational hierarchies, and diverse professional backgrounds in the surgical settings can further exacerbate challenges in verbal communication practices. To address these issues, incorporating digital communication platforms, encrypted messaging apps, mobile apps, real-time communication systems, and technological innovations into surgical workflows can expedite and streamline communication flow. This can improve decision-making and improve the surgical team’s efficacy, thereby reducing their workload and enhancing patient and patient caregiver experience in surgical settings.^9,10^ The incorporation of digital health communication technologies has transformed healthcare communication practices across all perioperative phases.^11-13^ Tailored digital communication interventions, ranging from (mHealth) applications to smartphone-based applications, allow for more personalised, accessible, and engaging communication among healthcare providers and patient caregivers. Despite the benefits of digital communication practices, challenges such as low digital literacy levels, data security and privacy concerns, inconsistent reporting quality, infrastructural limitations, medico-legal liabilities, and ethical, legal, and organisational concerns highlight the need for context-specific and user-centred innovations.^11-14^ The COVID-19 outbreak (WHO, 2020), has served as a catalyst for the rapid adoption of digital technologies at an unprecedented rate. While demonstrating the importance of digital communication tools, it also highlighted significant challenges in the widespread adoption of digital communication tools in diverse healthcare settings worldwide.^14-17^

Despite communication breakdowns being one of the primary causes of adverse events in surgical settings, literature gaps persist that hinder the development of best communication practices in these settings. Existing reviews have focused on improving healthcare communication practices in primary and acute care settings, including inpatient wards, intensive care units, and recovery rooms. There is limited evidence and research on communication practices in perioperative care.^18,19^ The evidence is skewed towards urban and high-resource surgical settings, leaving a significant research gap in various geographical and low- to middle-income surgical contexts. Published literature documents notably varied communication practices, ranging from unstructured verbal updates to advanced digital technological innovations, such as short messaging systems, electronic boards, audio-visual boards, and mobile applications, through quantitative studies.^20-26^ There is limited qualitative research focusing on the perceptions of patient caregivers and surgical staff regarding various communication practices in surgical settings, including both their benefits and challenges. These qualitative insights are essential for framing tailored and context-specific communication strategies, ultimately reducing patient caregiver stress, decreasing surgical staff workload, streamlining communication flow, and enhancing patient surgical outcomes.^24-26^ This qualitative study fills the knowledge gap by identifying various communication practices in surgical settings through the perceptions and experiences of healthcare providers and patient caregivers. Specifically, through the patient caregiver experiences, this study captures their needs and expectations regarding communication practices in perioperative settings. Also, it identifies the healthcare provider’s perspectives on communication practices and challenges within surgical environments. This can contribute to the development of more effective communication practices within surgical environments. Beyond identifying communication gaps, this study illustrates the tension between culturally rooted preferences for personalised verbal communication and the systemic constraints of high-volume teaching hospitals. Although traditional communication approaches are more associated with reinforcing trust and building empathetic connections with the patient caregivers, their operational feasibility is limited by resource-constrained contexts. This perspective contributes to a more nuanced explanation of existing communication inefficiencies.

### 1.1. Conceptual Framework


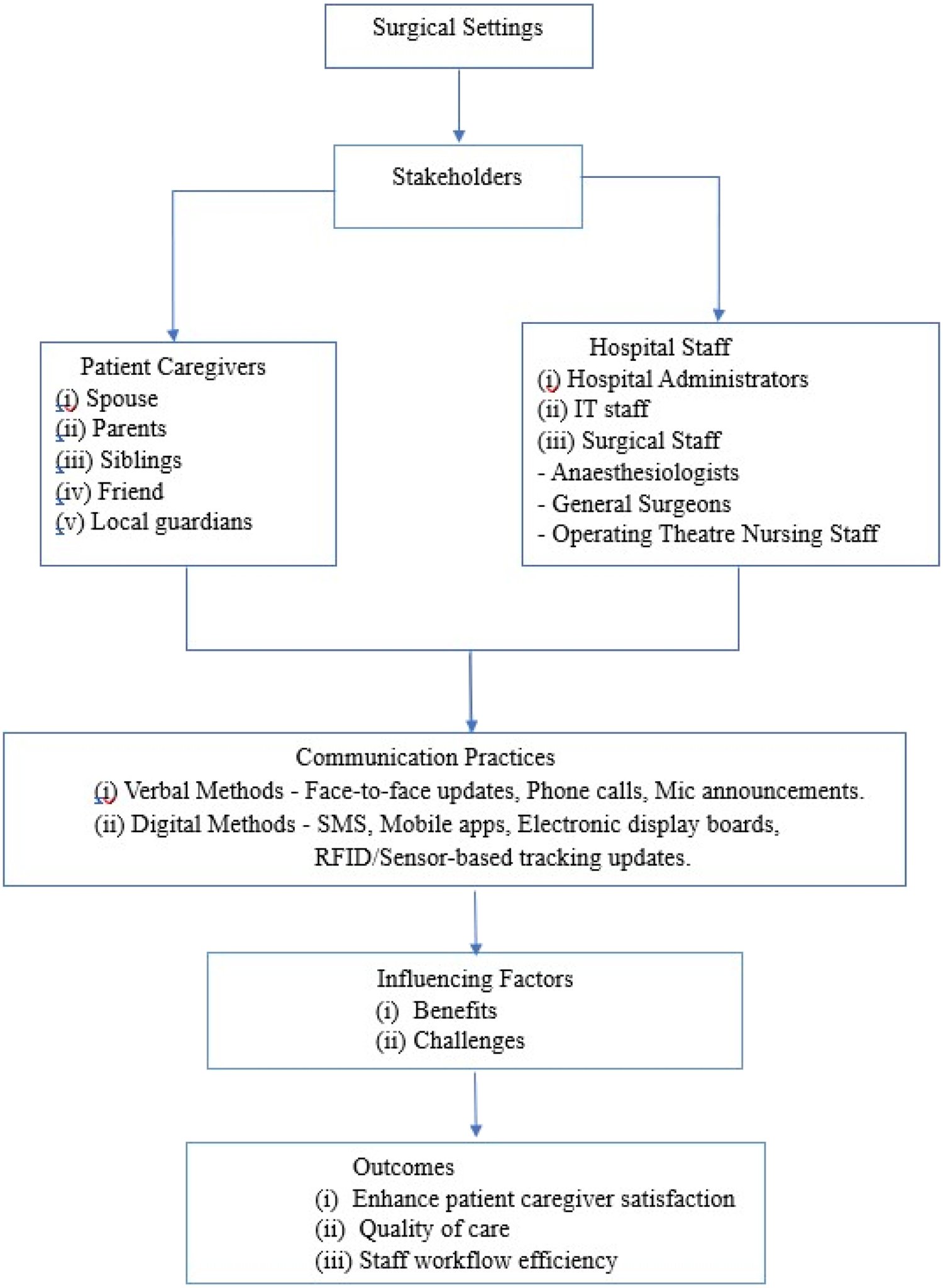


## 2. Methodology

### 2.1. Study Design

This research employs a qualitative approach to explore and understand the perspectives and experiences of various stakeholders concerning the different communication practices adopted in the surgical settings of a teaching hospital in coastal Karnataka, India. The qualitative study was undertaken over a three-month period, from May 2025 to July 2025. This methodological approach was chosen for its ability to gather rich, detailed insights that reflect participants’ perspectives in their own words. The two participant groups – healthcare staff and patient caregivers were purposefully included to capture a holistic understanding of communication practices from both the provider and recipient viewpoints, thereby pinpointing the potential communication gaps and mismatches within surgical settings. The utilization of in-depth interviews facilitated a nuanced exploration of the participants’ experiences, with the objective of identifying communication practices that enhance patient-caregiver satisfaction in surgical settings.

### 2.2. Data Collection Procedures

This study utilized a semi-structured in-depth interview guide to collect data. Two separate in-depth interview guides were prepared for two groups of stakeholders: one for healthcare staff and one for patient caregivers, to explore different communication practices in surgical settings. The interview guides, which were first developed in the English language, were later translated into Kannada, the local language of the study participants in Coastal Karnataka. The local bilingual researchers reviewed the translation process to ensure both linguistic accuracy and contextual appropriateness. A total of 23 key stakeholders participated, comprising 12 patient caregivers and 11 hospital staff members. The term patient caregiver in this context refers to a family member, such as a spouse, parents, siblings, in-laws, or family friend, who had accompanied the patients during surgery to provide care, emotional, informational, and logistical support, while waiting in the surgical waiting area throughout the perioperative period. These individuals, who comprised the patient caregiver group, played a direct role in perioperative communication at that given point in time. The participants were selected through purposive sampling from diverse professional backgrounds. Explicit inclusion and exclusion criteria were outlined for both groups.

Inclusion Criteria:

• Patient caregivers who accompanied a surgical patient and remained in the surgical waiting area throughout the perioperative phase.
• Individuals who were able to communicate in English and Kannada.
• Study participants aged 18 years and above.
• Healthcare staff who were either directly involved in the surgical workflows and patient care or involved in perioperative operational processes, such as administrators and IT personnel.

Exclusion Criteria:

• Individuals unwilling to participate voluntarily and declining consent.
• Hospital Interns and Trainees not primarily responsible for surgical communication processes.

In-depth interviews were conducted for all stakeholders. Patient caregivers and surgical staff were interviewed in the closed counselling room of the surgical theatre, while hospital administrators and IT department staff were interviewed at mutually convenient times and locations within the hospital premises. The data collection took place from May to June 2025, with each interview lasting approximately 40 to 50 minutes. Study participants provided informed consent, confirming their voluntary participation and understanding of the study’s aims and procedures. Written informed consent was obtained from all study participants before the commencement of data collection. Participants were informed about the purpose of the study, data collection procedures, the voluntary nature of participation, and the right to withdraw from the study at any stage without any consequences. Signed consent forms were maintained securely in compliance with institutional ethical requirements. An interview guide was developed based on the research questions and objectives. This was further content-validated through a pilot study. Open ended questions were asked to elicit responses from study participants about their own perspectives and experiences related to communication practices in surgical settings. All interviews were audio-recorded, transcribed verbatim, and stored securely to maintain confidentiality throughout the research process.

### 2.3. Data Analysis

The interview transcripts were thematically analysed using ATLAS. ti. version 8 software following a two-cycle coding process (Saldana, 2011). A set of deductive codes was developed in the first stage, based on the conceptual framework of the study, which primarily focused on various communication practices, the experiences of both stakeholder groups, the enabling factors, and their subsequent impact. A set of inductive codes was generated directly from the narratives and experiences of patient caregivers and healthcare providers, thus capturing unanticipated insights on various communication practices and their benefits and challenges in surgical settings. Through this structured process, a total of eleven codes were developed. These codes were further categorized and merged into code groups or code families during the second stage of analysis. This entire process led to the development of six overarching themes: communication practices, verbal communication methods, verbal communication challenges, digital communication tools, digital communication benefits, and digital communication challenges. These themes reflected the perspectives of healthcare providers and patient caregivers. They have been presented in the Results section, supported with illustrative quotations from the participants. These themes collectively summarize the significant insights of the participants’ experiences with communication practices in surgical settings. Data coding and thematic interpretation involved an iterative and collaborative analytical process, with themes developed through researcher discussions and consensus-based agreement. Therefore, a former inter-rater reliability assessment using Cohen’s kappa was not conducted. The credibility of the findings was strengthened through data triangulation by incorporating diverse perspectives from multiple stakeholder groups, including patient caregivers and healthcare providers.

### 2.4. Ethical Considerations

Approval for this study was granted by the Institutional Ethics Committee on 24 January 2025 (IEC2 471/2024). Ethical approval was obtained from relevant administrative authorities at the study site. The study participants were provided with detailed information about the study’s aim and objectives, data collection procedures, and their rights. All data was anonymized to protect the privacy and confidentiality of study participants, who were also informed that they could choose to withdraw from the study at any given point without facing any consequences.

### 2.5. Trustworthiness of the Study

To enhance the trustworthiness of the study, measures focusing on criteria such as credibility and transferability of the research were undertaken. Pilot testing was conducted for the interview guide to ensure the comprehensibility and relevance of the questions. Rich, detailed, contextual descriptions of participants’ experiences were provided to maintain the transferability of the research. The reliability of the study was strengthened through pilot testing of the interview guide and verbatim transcription of all interviews, thereby ensuring the rigour and trustworthiness of the study. Utilisation of ATLAS. ti. version 8 software helped maintain a systematic coding process with iterative reviews, while an audit trail that documented all the methodical decisions supported the confirmability of the study. The transferability was reinforced by providing a comprehensive description of the study context along with participant narratives. Data saturation was attained after 23 interviews, beyond which no new themes emerged. The researcher acknowledges their positionality within the study setting and its potential influence on the participants’ responses, particularly among healthcare providers. Measures were taken to reduce potential power dynamics by ensuring voluntary participation and confidentiality. The interviews were conducted in a neutral and unbiased manner, using open-ended questions to allow participants to express their opinions freely and to avoid influencing their responses. Reflexivity was maintained throughout the research process.

## 3. Results

This study included 11 participants from the hospital staff group, ranging in age from 27 to 58 years, with diverse professional and educational backgrounds, and a predominance of women (Table 1). The participants in this group represented a diverse range of professional backgrounds and responsibilities within the particular healthcare study setting. The participants belonged to the Hospital Operations department (2), the General Surgery department (2), the Anaesthesiology department (2), the Information Technology department (2), and the Nursing Services department, which had 3 participants: 1 Operation Theatre (O.T.) Nursing In-charge, 1 O.T. Assistant Nursing Supervisor, and 1 O.T. Nursing Staff. These study participants had a diverse range of professional experience, spanning from 1 year to 26 years in the current study setting. Including participants from diverse professional roles provided a comprehensive view of communication practices and operational responsibilities in surgical settings.

**Table 1.** Participant Profile: Hospital Staff

Partici- pant ID	Age (yrs)	Gender	Professional background	Roles & Responsibilities	Work experience
P1	29	F	Associate Manager Operations	Handling the administrative and operational activities of the Operating Theatres of the Kasturba Hospital, Manipal	2.5 yrs
P2	47	M	Associate Director Operations	Handling the entire administrative operations of the Kasturba Hospital, Manipal	19 yrs
P3	51	F	OT Nursing staff	Announcing surgical updates on the Mic in the Main O.T.	7 yrs
P4	56	F	OT In-charge	Handling staffing and patient care activities in the Main OT	4 yrs
P5	55	F	Assistant Nursing Supervisor	Handling staffing and patient care activities in the Main OT	1 year
P6	27	M	Gen. Surgeon	Dealing with patients of general surgery	1 year
P7	36	M	Gen. Surgeon	Dealing with patients of general surgery	9 yrs
P8	42	F	Anaesthesiologist	Dealing with Anaesthesia and Perioperative Care	9 yrs
P9	35	F	Anaesthesiologist	Dealing with Anaesthesia and Perioperative Care	4 yrs
P10	58	M	IT Dept	Handling the entire IT operations of the Kasturba Hospital, Manipal	26 yrs
P11	49	F	IT Dept	Working in the IT department as a software developer	26 yrs

This study comprised 12 patient caregivers, ranging in age from 21 to 57 years (Table 2). The majority of patient caregivers were women from a wide spectrum of family and social relationships with patients. The patients associated with these patient caregivers had surgeries that ranged from routine to complex: gall bladder surgery, laparoscopic surgery, biopsies, oncological surgery, decompressive craniectomy, hysterectomy, orthopaedic interventions, and thyroid surgeries. The heterogeneity in the patient-caregiver relationships and patients’ surgical conditions provided a multifaceted perspective of perioperative communication in surgical waiting areas.

**Table 2.** Participant Profile: Patient Caregivers

Partici- pant ID	Age (yrs)	Gender	Relationship to patient	Purpose of hospital admission
P12	25	F	Father	Onco-surgery
P13	21	F	Family friend	Orthopaedic Surgery
P14	43	M	Husband	Hysterectomy
P15	25	F	Father-in-law	Colon rectum operation
P16	26	M	Brother	Cyst operation in the throat
P17	29	F	Mother	Left eye lymphoma excision
P18	26	M	Father	Onco-surgery
P19	32	F	Father	Plastic Surgery
P20	41	F	Father	Decompressive Craniectomy
P21	40	F	Family friend	Gall Bladder Surgery
P22	57	F	Wife	Laparoscopic Surgery
P23	26	F	Mother	Partial thyroidectomy with lymph node Biopsy

The analysis resulted in the emergence of six major themes as listed below in (Table 3).

**Table 3.** Main Themes and Sub-Themes Extracted From the Data

Sl. No.	Themes	Sub-themes
1.	Communication Practices	(i) Current communication practices
		(ii) Previous surgical experience
		(iii) Limited comparative experience
		(iv) Contextual differences
2.	Verbal Communication Methods	(i) Verbal updates
		(ii) Mic Announcements
		(iii) Verbal communication preference
		(iv) Unexpected surgical events
3.	Verbal Communication Challenges	(i) Verbal communication challenges
		(ii) Patient Caregiver inconsistency
		(iii) Longer duration surgeries
		(iv) Manpower constraints
		(v) Restricted mobility
		(vi) Sensitive information handling
		(vii) Staff overload issues
4.	Digital Communication Tools	(i) Digital Communication Tools
5.	Digital Communication Benefits	(i) Digital communication benefits
		(ii) Flexible waiting experience
		(iii) Reduce patient caregiver anxiety
		(iv) Reduce staff burden
		(v) Operational benefits
		(vi) Selective information sharing
		(vii) Structured Communication flow
6.	Digital Communication Challenges	(i) Communication gaps
		(ii) Patient caregiver literacy gaps
		(iii) Financial challenges
		(iv) System Integration challenges
		(v) Digital Communication challenges

Table 3 provides a framework of the major themes and sub-themes related to the various communication practices employed in surgical settings.

To supplement the thematic analysis, recurring concepts identified across stakeholder narratives are presented below in Table 4.

**Table 4.** Frequently Emerging Concepts Across Participant Narratives

Concept	Narrative context
Verbal communication practices	Commonly discussed communication mechanisms in existing surgical settings
Mic announcements	Frequently reported communication approach
Patient caregiver stress and anxiety	Recurrently reported emotional responses
Digital communication perceptions	Repeatedly expressed need for communication enhancement strategies
Surgical waiting experience	Commonly associated with patient caregiver satisfaction and uncertainty regarding timely perioperative updates
Operational Challenges	Predominantly identified workflow-related challenges among healthcare staff

**Figure 1.**
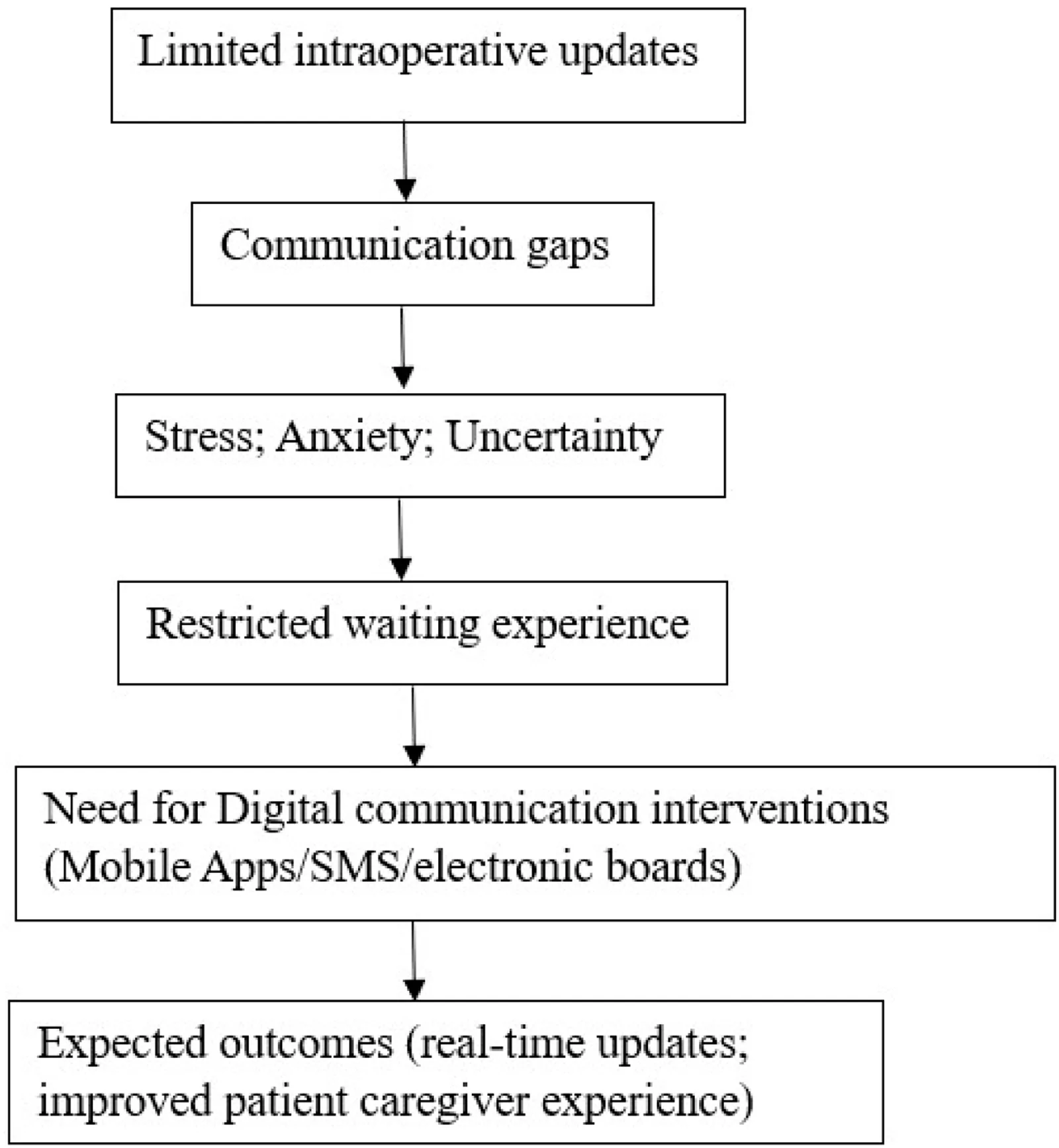
Conceptual representation of patient caregiver perspectives on communication practices in surgical settings

The thematic relationships identified from the patient caregiver interviews are illustrated in Figure 1, which depicts the conceptual representation of patient caregiver perspectives on perioperative communication practices.

**Figure 2.**
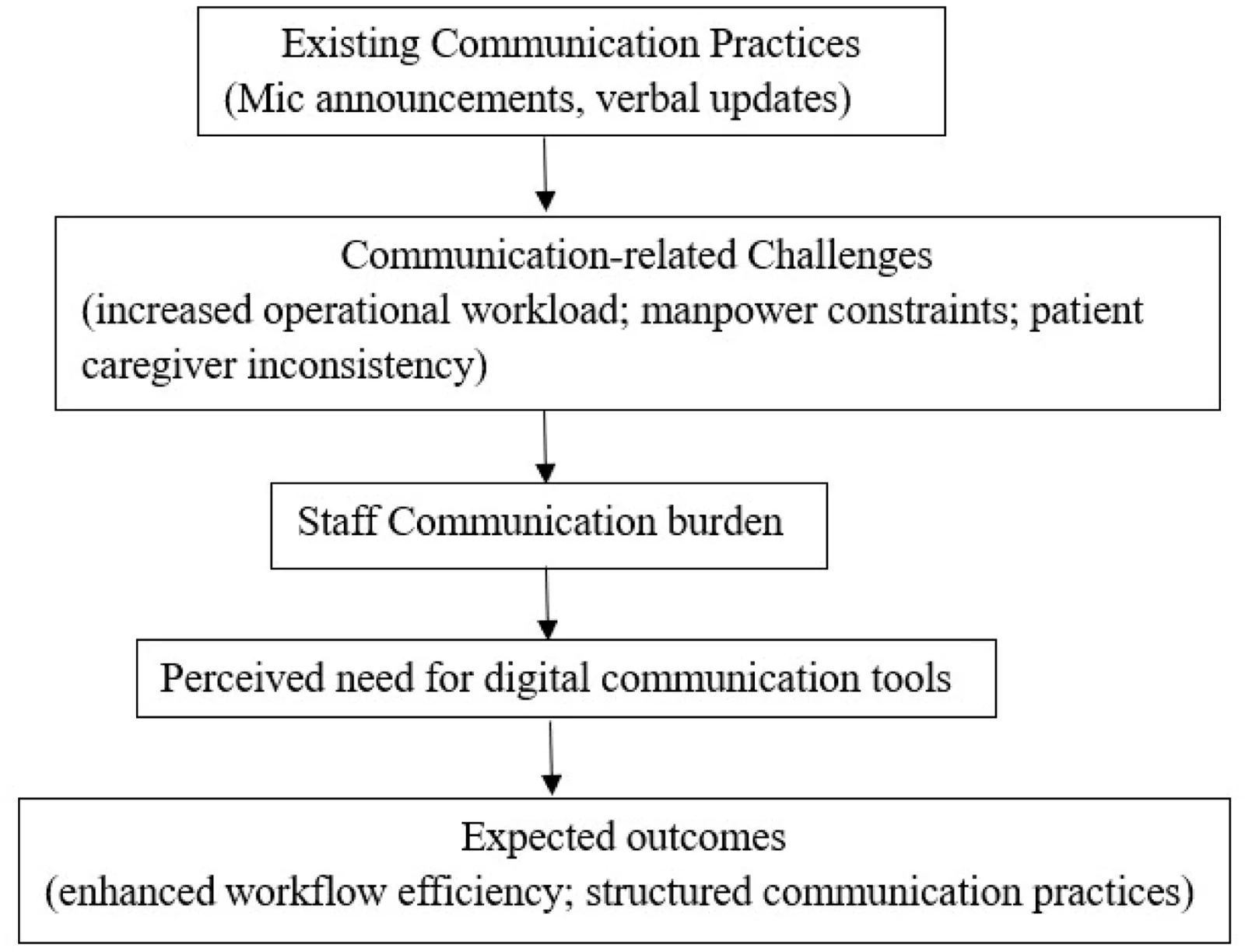
Conceptual representation of Healthcare Provider perspectives on communication practices in surgical settings

The thematic relationships identified from the healthcare provider interviews are illustrated in Figure 2, which depicts the conceptual representation of healthcare providers’ perspectives on perioperative communication practices.

Figures 1 and 2 provide conceptual representations of thematic relationships emerging from stakeholder experiences and illustrate the perceived impact of communication practices in perioperative settings.

### 3.1. Theme 1: Communication Practices

The healthcare providers described their current communication practices as primarily verbal, utilising a mic-based announcement system, and providing verbal updates during critical times. The surgical team, including the OT in charge, explicitly mentioned that patient caregivers are updated before surgery or once the patient is transferred back to the post-operative ward after surgery, but intraoperative updates remain limited.

“We inform the patient party to wait in the waiting area… in between any staff or communication means we will call through the mic system… So, right now we are practising this one.” (P4, 56, Female)

Hospital staff recognised that digital communication interventions would be more effective in corporate and non-teaching hospitals, where patient caregivers are updated regularly and are therefore better informed, compared to teaching hospitals which have intricate and sophisticated operational workflows and student involvement. Hospital staff also expressed concerns that implementing digital communication solutions might increase pressure on the surgical team if surgeries exceeded the expected time duration.

“While a digital tool might work well with the corporate setup… here in the teaching sector, I think it will be a little challenging process.” (P1, 29, Female)

The majority of the hospital staff shared limited experiences with alternative modes of communication practices in other healthcare settings, although one operational staff member acknowledged being aware of using digital electronic boards in an NABH–accredited hospital. Additionally, he noted that the previous attempt to use SMS-based updates in the current study setting was discontinued due to operational challenges.

“In other hospitals… there is one TV or big screen showing updates—patients in pre-op, surgery name, and so on. That is the way for NABH hospitals now. We tried once to send regular SMS formats about the patient, but somehow it failed… it was difficult for the nurse to keep updating the situation.” (P2, 47, Male)

In contrast, patient caregivers in the other group highlighted significant communication experiences in the context of surgical settings. Some patient caregivers mentioned that information gaps were more common in larger tertiary hospitals with higher surgical volumes compared to smaller healthcare facilities with lower surgical loads.

“In smaller hospitals… very few patients will be there. So, for them it is easy to communicate… but here patients are more… it is tough to communicate with everyone.” (P15, 25, Female)

Patient caregivers frequently highlighted the preponderance of verbal communication practices through verbal updates and mic announcements, emphasizing concerns about irregular, inconsistent, and infrequent surgical updates. They expressed frustration over the lack of intraoperative information, which resulted in a lack of clarity and transparency, leading to prolonged periods of uncertainty.

“Just they went OT, after that… we didn’t get any call. Now it’s 11 o’clock and I haven’t received any update. We have to wait till [the patient] reaches post-op. We know we are not going to get anything in between.” (P20, 41, Female)

Most patient caregivers demonstrated unfamiliarity with alternative communication tools in other surgical settings. Some patient caregivers shared both positive and negative experiences during previous surgical experiences across different time frames and surgical settings, ranging from empathetic staff interactions to dissatisfaction with insufficient intraoperative updates.

“Other hospitals? No. We have been here… for any surgeries, we have always been here. I have not experienced such digital tools. only here, like announcements.” (P21, 40, Female)

“My mother had a squamous carcinoma surgery… nearly 10 to 11 hours… I was going here and there asking them… they didn’t inform me of anything.” (P17, 29, Female)

### 3.2. Theme 2: Verbal Communication Methods

The surgical staff in this group commented that mic announcements and face-to-face verbal updates were the primary modes of communication used to inform patient caregivers in the existing surgical settings. The operating theatre nursing staff explained that although the patient caregivers were kept informed, it also created confusion, as it co-existed in two locations: the operating theatre reception area and the post-operative ICU.

“In between any staff or any communication means, we will call through the mic system in the reception area… such and such a patient party, please come to the reception counter” (P4,56, F).

“Sometimes I’ll ask security because post-op ICU also they’ll call, announce… then they’ll come directly here” (P3, 51, F).

One anaesthesiologist explained that it became difficult to provide real-time updates and maintain transparent communication during unexpected surgical events, as surgical complications could disrupt the flow of information.

“If something goes wrong during induction or intubation… we may have already informed relatives that surgery is going well, but suddenly things change, and that creates panic” (P8, 42, F).

Despite these limitations, the surgical staff believed that verbal communication practices were the most effective approach, as they maintained a personal connection with patient caregivers, making it a more pragmatic and culturally acceptable method. Some surgical staff were sceptical about the usability of digital communication tools in the current surgical context, as they felt that patient caregivers may not be technologically competent and may also be reluctant to accept impersonal modes of communication.

“Everything is explained verbally and consent is taken pre-op…Most of the patients here are from villages… I feel verbal would be easier” (P6,27, M).

From the patient caregiver’s standpoint, communication in the existing surgical context occurred either through public announcements, where their names were called over the mic, or through verbal updates from doctors, nursing staff, or security personnel.

“Yeah, mic, first of all, mic, they announced… only name, ‘Manjunath party, please come here” (P21,40, F).

“Through the security guards only. Once the operation is finished, they communicate through the guard to come and see the patient” (P15, 25, F).

Moreover, verbal communication was largely accepted and trusted by patient caregivers due to its cultural relevance. They preferred direct doctor-patient interaction over mediated communication platforms.

“People don’t trust digital means of communication as much as they trust physical means of communication” (P18, 26, M).

They also pointed out communication gaps, like receiving delayed and inconsistent updates, especially during unexpected surgical events, which made them more anxious and emotionally distressed.

“Morning, the patient went inside, and we don’t know anything… they’ll get scared” (P23, 26, F).

“Once they go inside the OT for one hour at least, we won’t know what is going on… we’ll be very anxious to know if there is any complication” (P17,29, F).

### 3.3. Theme 3: Verbal Communication Challenges

The healthcare providers in this study group consistently emphasised how verbal communication practices in surgical waiting areas are hindered by operational, systemic, and contextual factors, including interpersonal dynamics. Inconsistency among patient caregivers became a significant barrier to effective verbal communication practices, as the surgical team had to interact with different patient caregivers at various stages of the surgical process. As one surgeon noted, it not only amplified the communication burden on the surgical team but also led to communication gaps due to inconsistent dissemination of information. Also, during longer and more complex surgeries, the operating theatre staff often face constant inquiries from emotionally stressed patient caregivers.

“Then it becomes difficult to explain them overall again everything. Because in the ward, somebody else is there, and to the pre-op waiting area, someone else comes” (P6,27, M).

“Usually, it so happens that one person is available on the day before the surgery and another person is available just before the surgery, and the person who is there in the post-operative time is also completely different… it becomes a hassle” (P7, 36, M).

“Yeah, if surgery goes long, in between, we will usually have patient relatives only come and ask, so in the morning, they have come, so they shift the patient, what is happening? They themselves will come and ask” (P4, 56, F).

Staffing constraints and excessive workloads emerged as ongoing concerns, as the existing surgical team was already overloaded with clinical duties and unable to provide consistent and timely surgical updates to patient caregivers, especially during emergencies and crisis situations, when existing personnel were often diverted to provide clinical care to patients. One anaesthesiologist recalled:

“We have one scrub nurse and one floor nurse for each theatre. Okay, you don’t have an extra person. They have enough work to do. You cannot be burdening them with this one thing as well” (P8, 42, F).

“During that time, I think giving an update becomes difficult” (P9, 35, F).

Patient caregivers have restricted access to the operating theatre complex, relying mainly on verbal announcements, which results in communication delays. Due to practical and ethical considerations, surgical staff are unable to deliver sensitive information to patient caregivers, making it difficult to strike a balance between transparency and confidentiality of communication.

“Ideally, we should not call the patient caregiver or anything orally, name should not be disclosed, but we are disclosing all the names and everything” (P2, 47, M).

Another challenge faced by patient caregivers was being physically present in the surgical waiting area to avoid missing important verbal announcements.

“So, we have to be there in that room. We should not go anywhere. So, we have to wait for them to come and call us. I think that is not convenient for the patient and also for us” (P12, 25, F).

“I have to be there in that waiting room, even if I have other work, I cannot do it, because anytime, at any moment, they may call you” (P14, 43, M).

“We are just sitting there without knowing anything about the patient’s condition and the operation… the first time they told us it is around 6 hours, but now they are telling us it is around 8 hours. So, we do not know anything about that” (P16,26, F).

Patient caregivers reported incidents where such fragmented communication led to increased confusion and repeated inquiries. Additionally, the tone and clarity of communication delivery were perceived as emotionally stressful, particularly when conveying sensitive information and critical updates, as they were irregular and unreliable.

“They command and they tell something like that would scare you… it’s better when they are friendly and explain” (P13, 21, F).

### 3.4. Theme 4: Digital Communication Tools

The healthcare providers suggested the implementation of a few digital communication tools such as digital or electronic boards, like the ones displayed at airports, to show patient movement during all the perioperative phases. These tools were perceived as convenient and pragmatic communication practices that would alleviate the communication burden on the surgical team as well as streamline operational workflows.

“You can make a digital board where each patient's name is there and their current status, just like in the departure lounge of the airport. That might be more feasible.” (P7, 36, Male).

An anaesthesiologist raised privacy and confidentiality concerns, laying more emphasis on using alternatives like hospital numbers, token numbers or codes rather than publicly disclosing patient identifiers.

“No, we are not telling what surgery the patient is undergoing. We are just telling the patient's name, hospital number, and where the patient is currently.” (P6, 27, Male).

“Patient name we can’t display, some code words or something like this completed, like that.” (P5, 55, Female).

To facilitate seamless monitoring and real-time updates in surgical settings, the IT, operational, and administrative staff advocated a complete digital adoption of communication interventions.

“Once the data is captured in the electronic system completely, then implementing these kinds of solutions will be easy.” (P10, 58, Male).

The O.T. nursing staff highlighted several concerns regarding the practical implications of digital communication tools, including poor internet connectivity, limited digital readiness and access among some patient caregivers, and the potential of overburdening the surgical staff.

“Some people… will go with their mobile, so we have to call them repeatedly. And sometimes this internet problem is also there, so we can’t reach.” (P4, 56, Female).

“Maybe certain updates can be given like notifications… but if they keep on asking, it will be a hindrance to the surgeons.” (P9, 35, Female).

The patient caregivers suggested that digital communication tools should be more user-friendly and technically adaptable for populations across all age groups, such as mobile apps, electronic display boards, and SMS message updates in text or visual formats. Timely surgical updates were strongly preferred, with frequent updates every hour or every thirty minutes.

“People are comfortable using digital means of communication. App would be good, I think. Yes, I mean, a mobile app would be good.” (P23, 26, Female)

“Maybe at least once every half an hour… if in the mobile services, maybe it’s always, we want to know about them.” (P13, 21, Female)

“If that waiting person gets the proper update… then that person will not get stress, he will not be getting tension.” (P16, 26, Female)

The patient caregivers also valued the importance of having a flexible waiting experience where real-time updates would enable them to step out for refreshments or errands without stressing about missing critical updates.

“You don’t have to be in that waiting room only; you can also do other things.” (P14, 43, Male)

One patient caregiver reflected that implementation of digital communication interventions would yield collective benefits at a broader level, especially by alleviating congestion and reducing the chaotic atmosphere in the surgical waiting areas.

“It would be much less chaotic… congestion here also reduces… patient caregivers already stressed.” (P20, 41, Female)

### 3.5. Theme 5: Digital Communication Benefits

The hospital staff members suggested that digital communication interventions could potentially ease the communication burden on staff. This would help to improve operational efficiency and create a structured flow of communication. A hospital operations staff member acknowledged that digital communication platforms would generate real-time data on theatre utilization metrics, surgical delays, raw data on resource use, including the surgeon time utilization, block time utilization, case throughput, and surgical productivity, thus facilitating administrative oversight.

“From the administration perspective… whether OT is being utilized enough or not… with this application, we might be able to question why the same surgery is going beyond expected timelines” (P1,29, F).

An anaesthesiologist noted that the digital communication system would help alleviate the communication burden on surgical team members, especially in high-volume surgical environments where verbal communication practices can become tedious.

“Maybe when it is a long surgery it will definitely be of help… then there will definitely be of help” (P8,42, F). Similarly, an OT assistant noted the practical advantage of automation: “Like if anyone comes, I am not supposed to go inside and see, that I can see online, and I can tell them” (P5,55. F).

The nursing OT in charge emphasized the potential for a structured flow of timely updates through digital communication tools, which would replace the current inconsistent and fragmented communication flow.

“Sometimes we have also missed, because the patient shifted to the ICU… so in between, we miss the patient party to give the message. It will be helpful if you bring this step” (P4,56, F).

An anaesthesiologist reflected that sharing selective information through digital communication systems was beneficial, striking the right balance between transparency and sensitivity. This would also help ensure that critical updates were directly explained by doctors, thereby strengthening effective delivery of quality care and patient-centeredness.

“So I think that way, digital platform is a good option… it is one step towards improving quality of care to the patient, and that includes the patient caregiver” (P7,36, M).

The patient caregivers viewed digital communication practices as mechanisms that would help reduce their perioperative stress and uncertainty, especially during long and complicated surgeries. It would provide a flexible waiting experience where they receive real-time notifications.

“If it is apps or something, like any notifications you can get, you do not have to be in that waiting room only, you can go finish your work… it will enhance your waiting experience” (P14, 43, M).

“Because as you said, we are getting anxiety or stress when they enter the operation theatre. If we get the digital information, so it will be helpful to us” (P17, 29, F).

Another patient caregiver noted that digital communication platforms in the form of visual or textual formats would provide a pathway for a more structured, clear, and consistent dissemination of surgical information, alleviating the confusion and anxiety created by ad hoc verbal updates and manual announcements.

“Benefits is like it could maybe make the attenders less anxious because they know where the patient is and what is happening” (P20,41, F).

“Now, we do not have any information about patient condition, but if we have any apps, so we can see all the updates. Easily we will understand what is going on” (P16, 26, F).

“Text is enough… I just get to know that things are going fine till now, it is just a good thing” (P21, 40, F).

### 3.6. Theme 6: Digital Communication Challenges

Despite the benefits of digital communication practices in surgical settings, the hospital staff also pointed out a few limitations such as unreliable networks and connectivity issues, which highlighted the uncertainty that might come with over-dependence on digital technology in critical operational areas of the hospital.

“If server issues happen, the whole communication process will collapse. At that time, we will again depend on mic announcements” (P10,58, M).

The operations staff noted that patient caregiver information demands vary between teaching hospitals and corporate or non-teaching hospitals, where more proactive and timely surgical updates are required from the surgical team in the latter case.

“No, like I said, that’s what, since it is a teaching sector here, many patients come from poor and low socio-economic backgrounds, so they often struggle to understand digital technology. On the other hand, patients and families in the corporate hospital—all are highly educated—are generally more familiar with medical terms or smartphones, which helps them understand and use these systems better.” (P1,29, F)

One general surgeon said that patient caregivers in the current surgical settings they cater to exhibit socio-economic status disparieties, cultural differences, linguistic barriers, age gaps, and digital literacy gaps, emphasising the need for more user-friendly innovations that align with their current demographics. Patient caregivers from the comparative cohort also shared similar experiences, thus reinforcing the need for a tailored and accessible communication approach with multilingual communication support.

“I personally feel it is too sophisticated to use them based on the social status of patients that we encounter. I don't think they will be able to use the app appropriately. So, it might make it a little bit more difficult for them.” (P6,27, M).

Another challenge observed by the O.T. nursing staff was that during unexpected surgical events and emergencies, if the surgery goes beyond the expected duration and if the digital communication tools prematurely notify the patient’s caregivers about the surgery completion, then it can heighten the frustration and stress levels among them. This can lead to mistrust and perceived unreliability in automated communication interventions. Additionally, a human interface would be required from the doctor’s end to communicate personally with the patient caregiver during such critical surgical events.

“Digital is okay for routine updates, but critical issues should still be discussed face-to-face by the surgeon” (P4, 56, F).

Another central theme noted by the operations staff with the adoption of digital communication intervention was the medicolegal concerns that may arise because of privacy and confidentiality risks associated with the public display and circulation of sensitive patient information through electronic display boards, mobile platforms, and SMS notifications.

“But if you had a central display, like you say, in the waiting area, but then that again, it becomes a bit of a problem because why should the other patient's relative know what's happening to my patient? It's a question of privacy.” (P8, 42, F)

An anaesthesiologist addressed a few other issues, such as manpower constraints and overburdened existing staff in the highly stressful and busy operating theatre settings, amplifying the resistance to technological adoption. This emphasises the need for training and having designated communication personnel in current surgical settings to facilitate the seamless integration of digital communication systems into existing operational workflows.

“It should not be like another app or another login… it should merge with what we already use; otherwise, it becomes extra work” (P7, 36, M).

“So you would want one staff member designated wherein he will, in person, both take the update and, you know, circulate the information to the respective phone numbers attached in case and all like. So, additional manpower, it depends on the setup, like I said already, depends on how many OTs you have. So, will you have a single manpower to do that, or will you have to post multiple people?” (P1,29, F)

The O.T. ANS highlighted a system integration challenge arising from interruptions in wearable-based digital tracking systems, such as RFID-enabled technology. When the same arm with the wristband is used for IV cannulation, the wristband must be removed, thereby compromising the continuity of real-time information and hindering the communication flow of intraoperative digital updates to patient caregivers and support teams.

“Once the patient enters the OT, the outer gown is removed, and only the surgical site is exposed under a green sheet. After surgery, the sheet is removed and the patient is shifted to post-op. The ID band is often set aside, removed due to IV lines—especially if a central line is placed or blood loss is significant.” (P5, 55, F)

Moreover, one operations staff member questioned the financial feasibility of deploying a digital communication tool in a resource-limited surgical setting that would require a high initial investment in terms of software procurement, staff training, logistics, and infrastructural adaptations. Without strategic planning and user-friendly design, such systems would result in poor adoption and communication failures.

“Financially, we must see whether the hospital can maintain this system in the long run… it is not just installation but continuous costs” (P2,47, M).

While patient caregivers showcased technological readiness, aiming to bridge the communication gaps in perioperative settings, they also pointed out a few drawbacks of digital communication implementation, such as limited accessibility to smartphones and the digital literacy required to operate these systems. Additionally, the risk of excluding people raises concerns around equitable access to the interventions.

“Maybe some might not be aware of using cell phone technologies very well.” (P13,21, F)

“Yeah, senior citizens, rural area. So, they do not know any English or anything but using mobile.” (P15,25, F)

Patient caregivers demonstrated the need for compassionate and reassuring verbal communication during complications and emergency crises. Owing to socio-economic diversity, financial constraints were another challenge highlighted by patient caregivers, reflecting the need for a convenient, user-friendly, and cost-effective implementation of digital communication practices in surgical settings.

“If the hospital charges extra for this facility, it will not be affordable for everyone” (P22,57, F).

## 4. Discussion

Effective communication in perioperative settings between healthcare providers and patient caregivers plays a crucial role in patient safety, quality of care, operational efficiency, and overall patient caregiver experiences.^6,15,19^ The study findings demonstrate that the existing communication practices in this teaching hospital of coastal Karnataka heavily depend on verbal communication in the form of face-to-face updates and mic announcements. Although this approach was more culturally accepted, widely adopted, and trusted, it still failed to provide consistent, timely, clear, and transparent communication, especially during complex, long surgeries, thereby heightening patient caregiver anxiety, stress, and uncertainty. The present findings also exhibited a complex roleplay between the benefits and challenges of both verbal and digital communication practices, from the perspective and experiences of stakeholders from both groups. Participants reported that verbal communication practices foster empathy, warmth, and human connection, especially among populations with diverse cultural backgrounds, socio-economic strata, age groups, linguistic profiles, low literacy proficiency, and rural contexts. However, this method highlighted several limitations, such as operational overload and manpower constraints during emergencies and prolonged surgeries, thereby increasing the staff communication burden and requiring timely information delivery. While the surgical staff observed constant patient caregiver rotation as one of the major drawbacks leading to disruptions in the continuity of information delivery, patient caregivers identified restricted mobility, limited access, confidentiality concerns associated with mic announcements, and a lack of intraoperative updates as drawbacks contributing to an inflexible waiting experience. Recent studies have supported these findings, reinforcing that perioperative communication is often limited by time and staffing constraints, thereby poorly impacting the staff-patient caregiver interactions, information delivery, and patient quality of care.^19,27,28^ Previous studies have linked fragmented communication, information gaps, insufficient teamwork, and collaboration among surgical staff in a high-stress surgical environment to contribute to (2.9%–3.7% of hospitalisations) adverse events, thereby compromising patient safety and trust in healthcare.^19,29^

The study participants perceived the implementation of digital communication tools such as electronic display boards, SMS updates, and mobile platforms as a means to bridge communication gaps, thereby providing structured and real-time communication. The surgical staff demonstrated the potential benefits, including optimising staff efficiency, minimising staff communication overload, enhancing team collaboration, promoting operational transparency, and providing operational theatre utilisation metrics to the administrative staff. These timely updates would help reduce patient caregiver emotional stress and anxiety, thereby improving their waiting experience in surgical contexts. System integration challenges, high implementation costs, unreliable network and connectivity issues, data security and privacy concerns, confidentiality issues about digital boards, low digital literacy, socio-cultural disparities, system breakdown leading to communication failure, medicolegal risks, resource constraints, and additional operational overload on staff were significant barriers to digital communication practices in existing surgical contexts. To strengthen surgical communication, a dedicated, trained personnel member can facilitate timely updates to patient caregivers and reduce the information burden on surgical staff, thereby streamlining communication pathways in surgical settings. Published research has corroborated the study findings that regular intraoperative text messages in surgical oncology patients have helped to reduce psychological stress and anxiety levels among patient caregivers.^19,24,25^ Similar findings were reported by an Iranian study, where both video training and intraoperative progress reports proved to be effective digital tools in reducing anxiety in patient caregivers.^19,30^ Two Randomised Controlled Trials implemented in Turkey showed a significant reduction in anxiety levels of patient caregivers when they were informed by short messages. One study examined the improvement in patient caregivers’ satisfaction levels during orthopaedic surgical interventions. Another study focused on cardiovascular surgeries, reporting lower anxiety scores in the experimental group compared to the control group (P < 0.001),.^19,31,32^ Nevertheless, prior research has also shown considerable improvements in the performance and operational efficiency of surgical team members, as well as patient-centred effectiveness, following the successful implementation of digital tools.^33-36^

The results provide significant insights into current communication practices in perioperative settings: interaction with patient caregivers is often inconsistent, fragmented and heavily dependent on surgical staff. A recurring theme is a lack of standardised, structured communication protocols, thereby compromising the quality and timeliness of information shared throughout the surgical phase. The findings also demonstrate that communication practices are shaped by underlying operational dynamics such as surgical loads, availability of theatre staff and patient caregiver heterogeneity. These limitations often hinder the ability of surgical staff to provide timely and comprehensive updates, thereby resulting in communication breakdowns. Although verbal communication practices remain indispensable for fostering empathetic and personalised interactions in time-sensitive and high-pressure environments, a limitation is over reliance on provider availability. Digital communication interventions are potentially more reliable, systematic, and prompt. Their successful implementation is contingent upon factors such as accessibility, ease of use, usability, and contextual appropriateness in varied clinical environments. Collectively, when the study findings are considered alongside previous research, they provide a broader evidence base for implementing digital communication interventions in surgical settings to enhance patient care, to improve the overall patient-caregiver experience and surgical team efficacy.

The thematic insights are best interpreted within established theoretical frameworks. The prominence of timely, comprehensive and accessible communication aligns with the core principles of patient-centred communication, which focus on the patient and patient caregiver requirements.^27,37-39^ The interlinkage between technology-based communication platforms, end users, and contextual limitations aligns with sociotechnical systems, highlighting the need for fitting technological solutions with human factors and existing operational workflows.^40^ The documented differences in patient caregiver comprehension and engagement underscore the relevance of health literacy in facilitating effective communication to intended stakeholders within perioperative settings.^41,42^ These perspectives strengthen the theoretical underpinnings of the research and position the findings within a broader conceptual discourse. The study findings substantiate the proposed conceptual framework by reinforcing the relationships between stakeholders, communication practices, influencing factors, and outcomes in surgical settings. The findings further shape the framework by demonstrating the effectiveness of different digital communication interventions and the impact of various contextual determinants, such as institutional workload and technological feasibility, on their successful implementation. They also provide empirical support to the framework by showcasing the dynamics between various communication approaches and influencing factors. This ultimately enhances the care quality and operational efficiency.

Several themes highlighted conceptual overlap, particularly between verbal communication challenges and the need to implement digital communication interventions in surgical settings. Emotional experiences across participant narratives primarily reflected negative sentiments, including anxiety, uncertainty, stress, frustration, and confusion. In contrast, positive emotional reactions were associated with communication transparency, reassurance, and timely information sharing, thereby demonstrating the multidimensional impact of communication practices on stakeholder experiences.

The identified digital communication tools demonstrate various features, advantages and limitations, thereby indicating the need for more context-specific adoption and implementation. They are summarised in (Table 5).

**Table 5.** Comparative Overview of Digital Communication Tools

Digital tools	Key feature	Advantages	Limitations
Mobile Apps	Real-time updates	Regular and prompt updates.	Technology –dependent: Digital literacy and access requirements.
Short Message Service (SMS)	One-way notifications	Widely accessible and User-friendly.	Restricted interaction due to its unidirectional nature.
Electronic display boards	Centralised display system	Useful in high-volume, busy environments; supports mass information sharing.	Privacy risks, Confidentiality concerns.
RFID system	Automated tracking	Real-time patient tracking system,	High implementation cost, Infrastructure dependent.

### 4.1. Limitations of the Study

This qualitative study has a few limitations. It was conducted in a single healthcare setting in a teaching hospital with a high surgical load, thereby restricting the generalizability of the results. The sample size may not include a broad spectrum of stakeholders’ experiences involved in surgical communication practices. Considering the exploratory qualitative nature of the present study, formal sample size or statistical power calculations were not performed. Instead, participant recruitment and sample adequacy were determined through purposive sampling and thematic saturation principles. Data generated from the self-reported responses of study participants may have introduced call bias and social desirability bias, as the presence of the researcher during the interview may have influenced them to critically highlight their current communication practices. The complexity and nature of the participant responses may have been affected by heterogeneity in experiences with digital communication interventions, staff workload pressures, and limited technological exposure. The findings on digital communication interventions are based on participants’ perceived expectations rather than their own lived experiences, thereby limiting their generalizability and practical applicability to the current study settings. No observational data was captured on the long-term effects of digital communication practices on patient-caregiver satisfaction levels. This would have provided a nuanced and comprehensive understanding of real-time communication and communication dynamics in perioperative settings.

## 5. Conclusions

This study elaborates on the need to refine communication practices in high-pressure, time-sensitive surgical settings. While verbal communication methods are culturally trusted and provide a human interface, over-reliance on them fails to deliver timely, structured, and consistent information dissemination, especially during complex and long duration surgeries. Digital communication tools offer promising solutions to provide real-time updates, enhance the patient-caregiver waiting experience, and improve staff operational efficiency. But their implementation is limited by socio-economic and ethical considerations, as well as technological barriers. Therefore, a hybrid communication approach tailored to the needs of stakeholders would help to bridge communication gaps in surgical settings. Collectively, the research underscores the importance of complementing traditional communication practices with technology-enabled communication models to achieve a reliable and efficient information delivery system. These standardised communication strategies will help to reduce the patient caregiver concerns and strengthen their overall surgical experience, while providing the hospital administrators and organisational decision-makers with a more patient-centric, efficient, and scalable communication framework within perioperative contexts.

### 5.1. Recommendations

Future research should focus on developing hybrid communication models, advancing user-friendly technical innovations, and conducting periodic training and feedback mechanisms for surgical team members and patient caregivers. This collectively can facilitate the seamless integration of cost-effective digital communication systems into existing operational workflows, promoting equitable access across diverse healthcare environments. Digital communication approaches may help address literacy-related barriers among patient caregivers in rural and low-resource settings by adopting economical, feasible, and widely used platforms such as WhatsApp and audio-based messaging systems. These studies should focus on conducting longitudinal assessments to evaluate the effectiveness of digital communication implementation in perioperative settings, providing valuable insights into effective healthcare delivery over an extended period and offering a potential trajectory for policy-making aligned with global sustainable healthcare goals.

### 5.2. Implications for Practice, Policy and Future Research

Hospitals adopting hybrid communication models should ensure adequate staff training, streamlined operational processes, and clear communication protocols to promote patient caregiver satisfaction. Careful consideration should be given to equitable access for all patient caregivers, especially those from marginalised and rural populations, older adults and individuals with limited digital literacy skills, as they may require alternative or supportive communication channels. Policy initiatives should strengthen inclusive digital communication strategies. Future research should evaluate the implementation, scalability, and equity outcomes of such models.

## Data Availability

The raw data supporting the conclusions of this article will be made available by the authors on request.*

## References

[1] Etheredge HR Fabian J . Communication in Healthcare: Global challenges in the 21st Century. Hamostaseologie. 2022;42(1):29-35. doi:10.1055/a-1685-7096. 34991176

[2] Nedelcu CM Niculiǎ OO Nedelcu V , et al. Effective communication and patient safety among nurses in perioperative settings: a best practice implementation project. JBI Evid Implement. 2022;20:S3-S14. doi:10.1097/XEB.0000000000000316. 36372788

[3] Causes R Type E . Sentinel Event Data 2004 – 2013. Published online 2013.

[4] The Joint Commission . Sentinel event data 2023 annual review. The Joint Commission. 2024:1-9.

[5] Carvill M Butler G . The power of effective communication. Can Marketing Save the Planet. 2024:130-131. doi:10.5040/9781399411226.0056.

[6] Garrett JH . Effective Perioperative Communication to Enhance Patient Care. AORN J. 2016;104(2):111-120. doi:10.1016/j.aorn.2016.06.001. 27472971

[7] The Joint Commission . National Patient Safety Goals Effective January 1, 2016. Hospital Accreditation Program. The Joint Commission. 2016:1-17.

[8] Osborne-Smith L Kyle Hodgen R . Communication in the Operating Room Setting. Annu Rev Nurs Res. 2017;35(1):55-69. doi:10.1891/0739-6686.35.55. 27935774

[9] Etherington N Wu M Cheng-Boivin O Larrigan S Boet S . Interprofessional communication in the operating room: a narrative review to advance research and practice. Canadian Journal of Anesthesia. 2019;66(10):1251-1260. doi:10.1007/s12630-019-01413-9. Springer New York LLC. 31140044

[10] De La Cruz Monroy MFI Mosahebi A . The Use of Smartphone Applications (Apps) for Enhancing Communication With Surgical Patients: A Systematic Review of the Literature. Surg Innov. 2019;26(2):244-259. doi:10.1177/1553350618819517. 30602332

[11] Espin S Indar A Gross M Labricciosa A D’Arpino M . Processes and tools to improve teamwork and communication in surgical settings: A narrative review. BMJ Open Qual. 2020;9(2):1-9. doi:10.1136/bmjoq-2020-000937. PMC7304801 32554445

[12] Bol N Smit ES Lustria MLA . Tailored health communication: Opportunities and challenges in the digital era. Digit Health. 2020;6:1-3. doi:10.1177/2055207620958913. PMC7520919 33029355

[13] Nanah A Bayoumi AB . The pros and cons of digital health communication tools in neurosurgery: a systematic review of literature. Neurosurg Rev. 2020;43(3):835-846. doi:10.1007/s10143-018-1043-0. 30334173

[14] Wang Z Tang K . Combating COVID-19: health equity matters. Nat Med. 2020;26(4):458. doi:10.1038/s41591-020-0823-6. 32284617

[15] Brooke BS Slager SL Swords DS Weir CR . Patient and caregiver perspectives on care coordination during transitions of surgical care. Transl Behav Med. 2018;8(3):429-438. doi:10.1093/tbm/ibx077. 29800402

[16] Dal MF Massaro M Rippa P Secundo G . The challenges of digital transformation in healthcare: An interdisciplinary literature review, framework, and future research agenda. Technovation. 2023;123:102716. doi:10.1016/j.technovation.2023.102716.

[17] Bidoli C Pegoraro V Dal Mas F , et al. Virtual hospitals: The future of the healthcare system? An expert consensus. J Telemed Telecare. 2025;31(1):121-133. doi:10.1177/1357633X231173006. 37226478

[18] Al-Shorbaji N . Improving Healthcare Access through Digital Health: The Use of Information and Communication Technologies. Healthcare Access. 2022. doi:10.5772/intechopen.99607

[19] Pandit DD Bhavana S Nileshwar A , et al. Global Communication Practices and Their Impact on Patient Caregivers’ Satisfaction in the Surgical Waiting Area: A Scoping Review. Healthcare (Switzerland). 2025;13(12):1-15. doi:10.3390/healthcare13121408. PMC12193462 40565437

[20] Wittenberg E Kerr AM Goldsmith JV . Family caregivers’ perceived communication self-efficacy with physicians. Palliat Support Care. 2021;19(5):540-546. doi:10.1017/S1478951520001236. 33239115

[21] Zota D Diamantis DV Katsas K , et al. Essential Skills for Health Communication, Barriers, Facilitators and the Need for Training: Perceptions of Healthcare Professionals from Seven European Countries. Healthcare (Switzerland). 2023;11(14):1-16. doi:10.3390/healthcare11142058. PMC10379454 37510499

[22] Ehrler F Rochat J Siebert JN Guessous I Lovis C Spechbach H . Use of a Semiautomatic Text Message System to Improve Satisfaction with Wait Time in the Adult Emergency Department: Cross-sectional Survey Study. JMIR Med Inform. 2022;10(9):e34488. doi:10.2196/34488. 36066921 PMC9490523

[23] Gutierrez MA Moreno RA Rebelo MS . Information and Communication Technologies and Global Health Challenges; 2017.

[24] Poudel RR Singh VA Yasin NF . The Effect of Intra-operative Text Messages in Reducing Anxiety Levels Among Family Members of Patients Undergoing Major Musculoskeletal Tumour Surgery. Indian J Orthop. 2020;54(2):208-214. doi:10.1007/s43465-019-00002-8. 32257039 PMC7096592

[25] Huang F Liu SC Shih SM , et al. Reducing the Anxiety of Surgical Patient’s Families Access Short Message Service. PMC1839580 17238576

[26] Mignault A Tchouaket Nguemeleu É Robins S Maillet É Matetsa E Dupuis S . Automated Intraoperative Short Messaging Service Updates: Quality Improvement Initiative to Relieve Caregivers’ Worries. JMIR Perioper Med. 2022;5(1):e36208. doi:10.2196/36208. 35436760 PMC9084444

[27] Kwame A Petrucka PM . A literature-based study of patient-centered care and communication in nurse-patient interactions: barriers, facilitators, and the way forward. BMC Nurs. 2021;20(1):1-10. doi:10.1186/s12912-021-00684-2. BioMed Central Ltd. 34479560 PMC8414690

[28] Yoo HJ Lim OB Shim JL . Critical care nurses’ communication experiences with patients and families in an intensive care unit: A qualitative study. PLoS One. 2020;15(7 July):5-7. doi:10.1371/journal.pone.0235694. PMC7347110 32645062

[29] Leong KBMSL Hanskamp-Sebregts M Van Der Wal RA Wolff AP . Effects of perioperative briefing and debriefing on patient safety: A prospective intervention study. BMJ Open. 2017;7(12):1-7. doi:10.1136/bmjopen-2017-018367. PMC5736045 29247103

[30] Bagheri M Maleki M Mardani A Momen-beromi MH Daliri S Rezaie S . The effect of video training and intraoperative progress report on the anxiety of family caregivers waiting for relatives undergoing surgery. Heliyon. 2022;8(8):e10065. doi:10.1016/j.heliyon.2022.e10065. 35992003 PMC9382265

[31] Baydemir S Metin Akten I . Effect of Informing Patient Relatives by Short Message (SMS) on Their Anxiety and Satisfaction Level During Orthopedic Surgical Intervention: A Randomized Controlled Trial. Journal of Perianesthesia Nursing. 2023;38(6):901-906. doi:10.1016/j.jopan.2023.02.008. 37665301

[32] Yılmaz S Calikoglu EO Kosan Z . for an Uncommon Neurosurgical Emergency in a Developing Country. Niger J Clin Pract. 2019;22(June):1070-1077. doi:10.4103/njcp.njcp. 31417049

[33] Ahmed A Ho CW Grant Y Archer S Carrington EV . Acceptability of digital health interventions in perioperative care: a systematic review and narrative synthesis of clinician perspectives. BMJ Open. 2025;15(3):1-13. doi:10.1136/bmjopen-2024-086412. PMC12010342 40090692

[34] Lam K Kinross JM . Digital health in surgery. The Digital Doctor: How Digital Health Can Transform Healthcare. 2025:323-338. doi:10.1016/B978-0-443-15728-8.00005-7

[35] Hoffmann C Kobetic M Alford N , et al. Shared Learning Utilizing Digital Methods in Surgery to Enhance Transparency in Surgical Innovation: Protocol for a Scoping Review. JMIR Res Protoc. 2022;11(9):e37544. doi:10.2196/37544. 36074555 PMC9501681

[36] Marwaha JS Raza MM Kvedar JC . The digital transformation of surgery. NPJ Digit Med. Nature Research. 2023;6(1):103. doi:10.1038/s41746-023-00846-3. PMC10232406 37258642

[37] Çakmak C Uğurluoğlu Ö . The Effects of Patient-Centered Communication on Patient Engagement, Health-Related Quality of Life, Service Quality Perception and Patient Satisfaction in Patients with Cancer: A Cross-Sectional Study in Türkiye. Cancer Control. 2024;31:10732748241236327. doi:10.1177/10732748241236327. 38411086 PMC10901059

[38] Annelie JS Lena H Sandra MAM Ewa CL Inger KH . The COMCARE Framework for Person-Centred Communication—A Practical Caring Framework. Scand J Caring Sci. 2025;39(3):e70074. doi:10.1111/scs.70074. 40619709 PMC12230379

[39] Garling KA Stewart MP . A new framework for elevating and updating clinical language: RAISED patient-centric communication. JAPhA Practice Innovations. 2024;1(1):100001. doi:10.1016/j.japhpi.2023.100001.

[40] Fitzpatrick PJ . Improving health literacy using the power of digital communications to achieve better health outcomes for patients and practitioners. Front Digit Health. 2023;5:1264780. doi:10.3389/fdgth.2023.1264780. Frontiers Media SA. 38046643 PMC10693297

[41] Cooper Z Cleary S Stelmach W Zheng Z . Patient engagement in perioperative settings: A mixed method systematic review. J Clin Nurs. 2023;32(17-18):5865-5885. doi:10.1111/jocn.16709. John Wiley and Sons Inc. 37073113 PMC10946744

[42] Seyfulayeva A Fonte BF Alho AM , et al. Patient and family engagement interventions for enhancing patient safety in the perioperative journey: A scoping review. BMJ Open Qual. 2025;14(1):e002986. doi:10.1136/bmjoq-2024-002986. BMJ Publishing Group. PMC11836844 39961679

